# ABNORMALITIES IN THE CITRATE CYCLE METABOLISM ARE ASSOCIATED WITH DECLINED INTRINSIC CAPACITY IN OLDER ADULTS

**DOI:** 10.1093/geroni/igac059.1285

**Published:** 2022-12-20

**Authors:** Yiming Pan, Pan Liu, Yun Li, Lina Ma

**Affiliations:** Xuanwu Hospital Capital Medical University, Beijing, Beijing, China (People's Republic); Xuanwu Hospital Capital Medical University, Beijing, Beijing, China (People's Republic); Xuanwu Hospital Capital Medical University, Beijing, Beijing, China (People's Republic); Xuanwu Hospital Capital Medical University, Beijing, Beijing, China (People's Republic)

## Abstract

**Background:**

Aging is accompanied by a decline in physical and mental functions, manifested as the declines in intrinsic capacity. However, there is still a lack of understanding about the metabolic mechanisms underlying the declining intrinsic capacity. Objective: To explore the metabolic characteristics and pathways of the declines in intrinsic capacity with the assistance of metabolomics methods.

**Methods:**

Our study recruited 38 participants in total. The Short Physical Performance Battery, Mini-Mental State Exam, 30-item Geriatric Depression Scale, self-reported hearing/visual impairment and Mini Nutritional Assessment were used to assess the five domains of intrinsic capacity respectively. The untargeted liquid chromatography-mass spectrometry-based metabolomics was performed on the serum of participants. Multivariate statistical analysis and pathway analysis were then implemented.

**Results:**

Among the 38 participants aged 66.50±15.20 years, 22 were with the declines in at least one domain of intrinsic capacity. In the 349 identified metabolites, 35 were candidate biomarkers for declining intrinsic capacity with variable importance in the projection > 1.5 and p < 0.05. Citrate cycle, tryptophan metabolism, phenylalanine and tyrosine metabolism, and Arginine biosynthesis were significant pathways associated with the declines in intrinsic capacity.

**Conclusions:**

Decreased intrinsic capacity is an age-related change with characteristic metabolomics features. Many pathways, especially the dysregulation of the citrate cycle metabolism, may be involved in the pathogenesis of intrinsic capacity decline.

